# In Vitro and In Vivo Biological Activity of Berberine Chloride against Uropathogenic *E. coli* Strains Using *Galleria mellonella* as a Host Model

**DOI:** 10.3390/molecules25215010

**Published:** 2020-10-29

**Authors:** Giulio Petronio Petronio, Marco Alfio Cutuli, Irene Magnifico, Noemi Venditti, Laura Pietrangelo, Franca Vergalito, Antonella Pane, Giovanni Scapagnini, Roberto Di Marco

**Affiliations:** 1 Department of Health and Medical Sciences “V. Tiberio”, University of Molise Via de Sanctis 3, III Ed. Polifunzionale, 86100 Campobasso (CB) Molise, Italy; giulio.petroniopetronio@unimol.it (G.P.P.); i.magnifico@studenti.unimol.it (I.M.); n.venditti@studenti.unimol.it (N.V.); laura.pietrangelo@unimol.it (L.P.); giovanni.scapagnini@unimol.it (G.S.); roberto.dimarco@unimol.it (R.D.M.); 2 Department of Agricultural, Environmental and Food Sciences (DiAAA), University of Molise, Via De Sanctis 3, III Ed. Polifunzionale, 86100 Campobasso, Italy; franca.vergalito@unimol.it; 3 Department of Agricultural, Food and Environment, University of Catania, Via S. Sofia, 100, 95123 Catania, Italy; apane@unict.it

**Keywords:** berberine chloride, UPEC, antimicrobial activity, *Galleria mellonella*, host–pathogen interactions

## Abstract

Berberine is an alkaloid of the protoberberine type used in traditional oriental medicine. Its biological activities include documented antibacterial properties against a wide variety of microorganisms; nonetheless, its use against *Escherichia coli* strains isolated from urinary infections has not yet been widely investigated in vivo. The emergence of antimicrobial resistance requires new therapeutic approaches to ensure the continued effectiveness of antibiotics for the treatment and prevention of urinary infections. Moreover, uropathogenic *Escherichia coli* (UPEC) has developed several virulence factors and resistance to routine antibiotic therapy. To this end, several in vitro and in vivo tests were conducted to assess the activity of berberine on uropathogenic *E. coli* strains. *Galleria mellonella* as an infection model was employed to confirm the in vivo translatability of in vitro data on berberine activity and its influence on adhesion and invasion proprieties of *E. coli* on human bladder cells. In vitro pre-treatment with berberine was able to decrease the adhesive and invasive UPEC ability. In vivo treatment increased the larvae survival infected with UPEC strains and reduced the number of circulating pathogens in larvae hemolymph. These preliminary findings demonstrated the efficacy and reliability of *G. mellonella* as in vivo model for pre-clinical studies of natural substances.

## 1. Introduction

Among plant secondary metabolites, alkaloids exhibit important pharmacological properties. Berberine is a quaternary benzylisoquinoline alkaloid of the protoberberine type isolated from the root, rhizome, and stem bark of many plant species such as *Coptis chinensis* Franch., *Berberis thunbergii* DC., *Hydrastis canadensis* L., and *Thalictrum lucidum*, that has a long history of use in traditional Chinese medicine [1]. This alkaloid is known to have therapeutic and pharmacological effects on cancer, malaria, gastroenteritis, diarrhoea, hyperlipidemia, diabetes, obesity, metabolic syndrome, hypertension, polycystic ovary and Alzheimer’s disease [2,3,4]. In vivo, animal studies and clinical studies have found low toxicity and few side effects of berberine [5]. Additionally, toxicity studies in vitro have shown that it has no significant genotoxic, mutagenic or cytotoxic activity [6,7]. Although berberine has demonstrated in vitro antibacterial action against several microorganisms, including bacteria, fungi, protozoa, trypanosomes and plasmodia, its use against uropathogenic *E. coli* strains is not yet adequately researched [8,9,10,11].

Urinary Tract Infection (UTI) occurs when the pathogens are able to colonize the urinary tract system, reaching more than 10^5^ CFU/mL in the urine [12,13]. It is estimated that 150 million UTI cases are reported each year worldwide with high social costs [14].

These infections are rare in boys and infrequent among 2- to 13-year-old girls [15]. The incidence of UTI increases significantly during adolescence; indeed, almost 33% of women have had at least one episode of UTI requiring antimicrobial therapy [16].

The main microorganisms found in UTIs are represented by Gram-negative bacteria including *E. coli*, *Klebsiella spp*., *Proteus spp*., *Pseudomonas aeruginosa*, and *Providencia spp*., while to a lesser extent, Gram-positive bacteria including *Staphylococcus spp*. and *Enterococcus faecalis* have been found [17,18,19,20]. Overall, the most common etiological agent for both uncomplicated and complicated UTIs is uropathogenic *Escherichia coli* (UPEC), as demonstrated by an epidemiological study conducted by Lauplande et al. that analyzed a total of 40,618 episodes of community UTI in which *E. coli* was the most common infectious microorganisms (70%) [21].

UPEC is a heterogeneous group of extraintestinal pathogenic *E. coli* with several virulence factors that allow these bacteria to colonize the urinary tract and resist the host’s defence mechanisms. These virulence factors are distinctive and very different from those observed in other human microbial communities, among them the type 1 fimbriae and P fimbriae are present on the bacteria surface [22,23,24,25]. As adhesive organelles, they enable bacteria to bind host cells and tissues, to resist the flow of urine and promote the invasion of urothelial cells. As well as promoting adherence, these virulence factors also stimulate UPEC invasion into the host epithelial cells, promoting UPEC survival in the urinary tract [26]. Moreover, UPEC can penetrate the umbrella cells that cover the bladder lumen, moving into the cytosol of the host cell and rapidly multiply. In this way, they can form intracellular biofilm communities that can contain several thousand bacteria protected from neutrophils and antibiotics action [27,28].

Antibiotics are the first-line treatment for urinary tract infections. In particular, complicated UTIs and symptomatic catheter-associated UTI (CAUTI) are treated with aminoglycoside with or without amoxicillin or second- or third-generation cephalosporin or broad-spectrum penicillin with or without aminoglycoside [29].

However, in recent years, high prescription rates of empirical therapies prescribed without antibiotic susceptibility testing have contributed to the development of multi-drug resistant pathogens, making the management of UTI more challenging [30,31]. Antibiotic resistance has led to a significant therapeutical challenge, so research and development for novel treatment strategies have become a pressing issue [32,33,34].

This phenomenon is not limited to UTIs but is a worldwide concern that has a profound impact not only on the medical field but also on social behaviour and economic implication [35].

For these reasons, in recent years, the scientific community has been focusing increasingly on folk medicine, and in particular, the use of plants in the treatment of infections [36]. Medicinal plants and their derivatives (i.e., phytoextracts) have been used since the beginning of the 21st century in the treatment of urinary infections, especially in defeating pathogen drug resistance [37,38,39]. The botanical drug mixture commonly used to treat UTIs depends on many factors, such as the portion of the plant used, as well as the method of phytoextract preparation. The main mechanisms of action include the antioxidant effect, the interference with UPEC’s ability to adhere and invade the urothelium and the antimicrobial action [40,41,42,43,44,45].

In this scenario, an in vitro study conducted by Boberek et al. demonstrated that berberine negatively regulates the FtsZ protein, which is critical to the viability and cell division of *E. coli* [46].

Moreover, this alkaloid selectively inhibits the synthesis and assembly of *E. coli* Pap fimbriae, a virulence factor that plays an important role in upper urinary tract infections [47,48]. Combined studies of microarray and proteomics have revealed that berberine concentrations below the Minimum Inhibitory Concentration (MIC) can alter several genes involved in membrane wall biosynthesis, transport and mobility [49].

More recently, the berberine inhibition action in biofilms formation by Quorum Sensing System alteration in a drug-resistant *E. coli* strain has been reported by Sun T. et al. [50]. Although there are several in vitro studies about berberine antibacterial properties [5], its anti-adhesive activity has not yet been thoroughly investigated by in vitro experiments on human cells and, above all, no studies demonstrate its efficacy against UPEC using in vivo models.

The Lepidoptera *Galleria mellonella* has been increasingly employed as an alternative to mammals in vivo models for microorganism pathogenicity studies, host–pathogen interaction and screening for testing new therapies [51]. Numerous antibiotics and/or substances with antimicrobial activity have been tested against a multitude of human pathogenic microorganisms using this alternative host model. Moreover, *G. mellonella* has provided advantages over the use of the murine model, including the possibility to test many bacterial strains in a limited time at low cost with easy insect management, this model is not intended to replace mammalian in vivo studies but is a powerful tool for preliminary screening of antimicrobial compounds [52]. In vitro assays are essential for the pre-screening of drugs; however, discrepancies are often observed between in vitro and in vivo experiments [52]. Despite the limitations of this model as the lack of adaptive immunity and detoxification systems found in mammals, the results obtained by *G. mellonella* are comparable to those obtained with animal models [53,54,55,56,57].

The study aimed to employ *G. mellonella* as an animal model to confirm the in vivo translatability of in vitro activity of berberine and its influence on the adhesion and invasion properties of four uropathogenic *E. coli* on bladder cells (5637 ATCC HTB-9).

To this end, two strains isolated from long-term catheters carriers’ patients and two purchased from international collections (*E. coli* ATCC 11775 and *E. coli* 22312 DSM) were tested by several in vitro and in vivo assays.

## 2. Results

### 2.1. Berberine MIC Determination

MIC values (μg/mL) for Berberine chloride against the tested *E. coli* strains ranged from 1024 to 2048 µg/mL. The highest MIC values were 2048 µg/mL for *E. coli* DSM 22312 and *E. coli* CL2. The lowest MIC values were observed for *E. coli* ATCC 11775 and *E. coli* CL1 (Table 1).

### 2.2. Influence of Berberine on In Vitro Bacterial Growth

**Figure 1:**
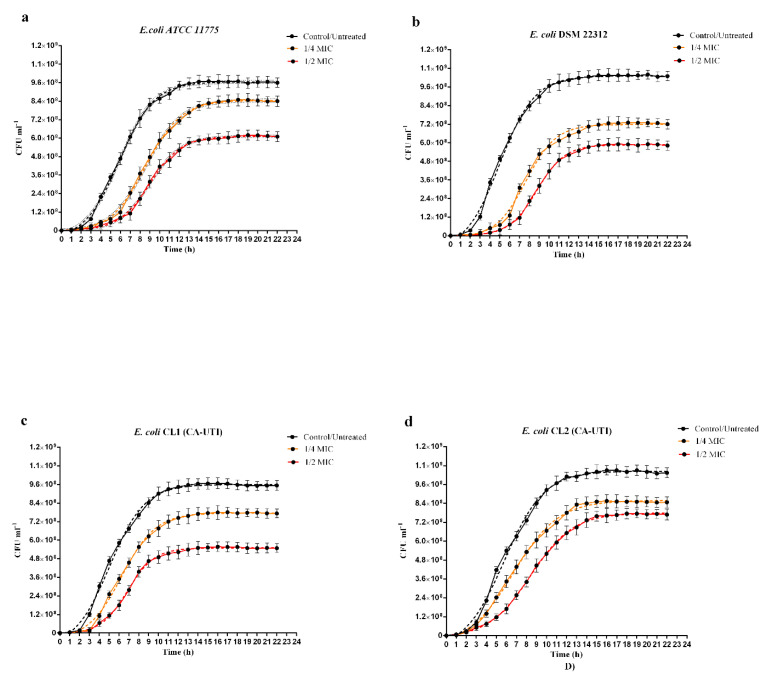
Influence of Berberine chloride on in vitro growth curves of *E. coli* strains by Cell Counting. Black continuous lines: untreated controls; black dashed lines: nonlinear fit of untreated controls; continuous orange lines: strains treated with berberine 1/4 MIC; orange dashed lines: nonlinear fit of strains treated with berberine 1/4 MIC; continuous red lines: strains treated with berberine 1/2 MIC; red dashed lines: nonlinear fit of strains treated with berberine ½ MIC. (**a**) *E. coli* ATCC 11775. (**b**) *E. coli* DSM 22312. (**c**) *E. coli* CL1 (CA-UTI). (**d**) *E. coli* CL2 (CA-UTI). The bars represent means ± SD of independent experiments performed in triplicate.

As shown in Figure 1 for all strains tested, the effect of berberine on in vitro growth and delay in reaching the exponential growth phase was both concentration and strain-dependent. As regards the inhibition rates (% inhibition), both concentrations of berberine reduced the in vitro growth in all tested strains. The highest percentage of inhibition was observed for *E. coli* DSM 22312 treated with 1/2 MIC of berberine (43.03%) (Figure 1b and Table 2) while the lowest percentage was observed for *E. coli* ATCC 11775 treated with 1/4 MIC (12.47%) (Figure 1a and Table 2). In all curves (Figure 1), a delay in reaching the growth exponential phase (∆X_CFU50_) was observed, the highest delay value was recorded for *E. coli* DSM 22312 (3.7 h) (Table 2) and the lowest for *E. coli* CL2 (0.9 h) (Table 2). The steepness of the curve (HillSlope) exerted by berberine was the slightest bit different for all strains. Indeed, berberine administration on *E. coli* ATCC 1775, *E. coli* DSM 22312 and *E. coli* CL1 showed a small increase in the steepness of the curve compared to the untreated control (Table 2), in contrast, for *E. coli* CL2, the hillslope values decreased (−0.03 for 1/4 MIC and −0.02 for 1/2 MIC) (Table 2).

### 2.3. Effect of Berberine on the Adhesion and Invasion of E. coli Strains to Human 5637 ATCC (HTB-9) Cell Line

Since adhesion and invasion are essential pathological mechanisms of *E. coli*, causing UTI, we investigated the impact of berberine chloride on UPECs interactions with the human bladder.

**Figure 2:**
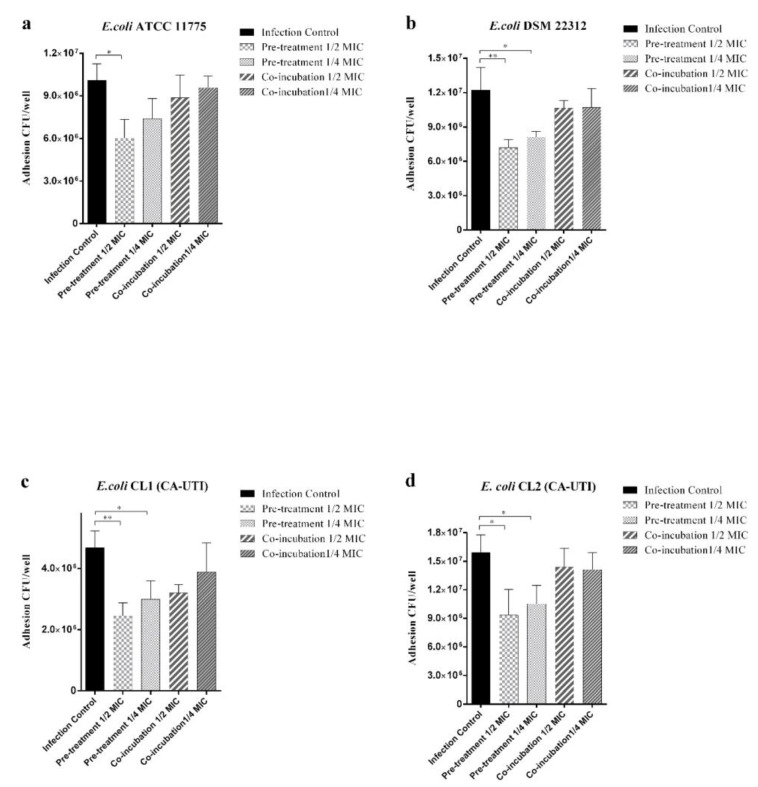
Effect of berberine on *E. coli* strains adhesion 5637 ATCC (HTB-9) cell line. Black histograms: strains not treated with berberine. Light grey histograms with large squares: strains pre-treated with 1/2 MIC berberine. Light grey histograms with small squares: strains pre-treated with 1/4 MIC berberine. Dark grey histograms with wide lines: strains co-incubated with 1/2 MIC berberine. Dark grey histograms with narrow lines: strains co-incubated with 1/4 MIC berberine. (**a**) *E. coli* ATCC 11775. (**b**) *E. coli* DSM 22312. (**c**) *E. coli* CL1 (CA-UTI). (**d**) *E. coli* CL2 (CA-UTI). Results are expressed as CFU/well. The bars represent means ± SD of three independent experiments performed in triplicate. Statistically significant differences, determined by one-way analysis of variance ANOVA (* indicates a significant difference at *p* < 0.05 versus control/untreated, ** indicates a significant difference at *p* < 0.01 versus control/untreated).

All bacterial strains not treated with berberine demonstrated a different tropism towards 5637 cells line with a mean of adhesion values of 1.01 × 10^7^ CFU/well for *E. coli* ATCC 11775, 1.22 × 10^7^ CFU/well for *E. coli* DSMZ 22312, 4.68 × 10^6^ for *E. coli* CL1 and 1.59 × 10^7^ CFU/well for *E. coli* CL2 (Figure 2). *E. coli* pre-treatment with 1/2 MIC and 1/4 MIC significantly reduced adhesion for three of four strains tested except for *E. coli* ATCC 1775 where only the 1/2 MIC concentration was statistically significant. The cells co-incubation with both berberine concentrations showed no significant effect on the adhesion ability for all of the tested strains (Figure 2).

**Figure 3:**
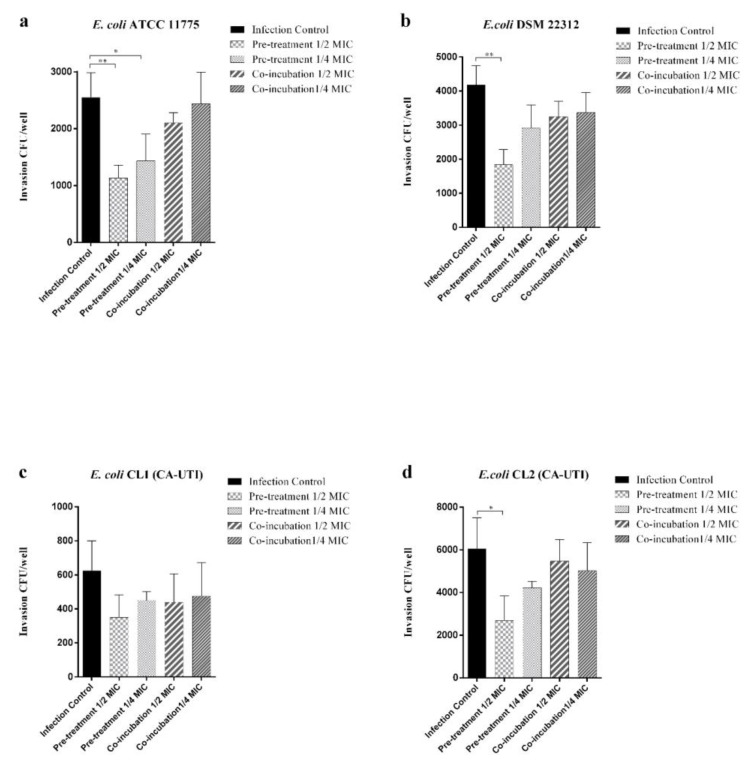
Effect of berberine on *E. coli* strains invasion ability against 5637 ATCC (HTB-9) cell line. Black histograms: strains not treated with berberine. Light grey histograms with large squares: strains pre-treated with 1/2 MIC berberine. Light grey histograms with small squares: strains pre-treated with 1/4 MIC berberine. Dark grey histograms with wide lines: strains co-incubated with 1/2 MIC berberine. Dark grey histograms with narrow lines: strains co-incubated with 1/4 MIC berberine. (**a**) *E. coli* ATCC 11775. (**b**) *E. coli* DSM 22312. (**c**) *E. coli* (CA-UTI) CL1. (**d**) *E. coli* CL2 (CA-UTI). Results are expressed as CFU/well. The bars represent means ± SD of three independent experiments performed in triplicate. Statistically significant differences, determined by one-way analysis of variance ANOVA (* indicates a significant difference at *p* < 0.05 versus control/untreated, ** indicates a significant difference at *p* < 0.01 versus control/untreated).

As shown in Figure 3, the pre-treatment with berberine 1/2 MIC significantly reduces the ability of *E. coli* ATCC 11775, *E. coli* DSM 22312 and *E. coli* CL1 to invade the bladder cell line. Pre-treatment with 1/4 MIC berberine significantly reduces invasive capacity only for *E. coli* strain ATCC 11775. As regards *E. coli* CL1, a non-significant reduction in invasion assay for both tested berberine concentration was observed. Co-incubation treatments, as well as adhesion assay, did not alter the invasive ability of the tested strains.

### 2.4. Infection Assays

**Figure 4:**
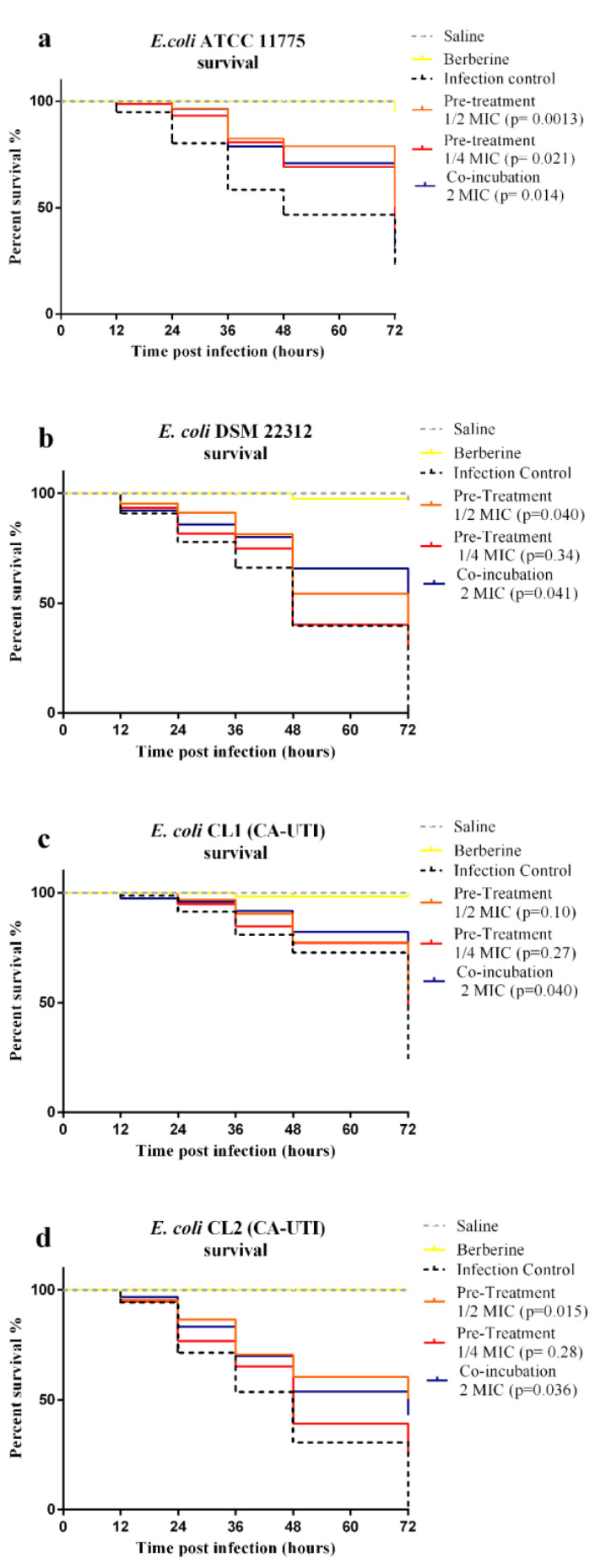
Kaplan–Meier survival plots of *G. mellonella* larvae by *E. coli* strains infection. Light grey dashed line: larvae inoculated with saline; Yellow line: Berberine toxicity control; Black dashed line: larvae inoculated with 10^5^ CFU/larvae; orange line: larvae infected with berberine pre-treated *E. coli* at 1/2MIC; red line: larvae infected with berberine pre-treated *E. coli* at 1/4MIC; blue line: larvae infected with *E. coli* and co-incubated with 2 MIC berberine. (**a**) *E. coli* ATCC 11775. (**b**) *E. coli* DSM 22312. (**c**) *E. coli* CL1 (CA-UTI). (**d**) *E. coli* CL2 (CA-UTI). All groups of each treatment were compared with the control group using the log-rank test; the *p* values are reported for each group.

To verify the berberine in vivo effects against UPEC, infection assays on *G. mellonella* larvae were carried out. Preliminary, the berberine toxicity at concentrations ranging from 4096 µg/mL to 64 µg/mL on uninfected larvae was assessed. No larvae mortality was observed (data not shown). *G. mellonella* larvae survival plots for the different treatments are presented in Figure 4.

The *E. coli* infection without berberine treatment caused the death of 76.64% larvae for *E. coli* ATCC 11775, 100% for *E. coli* DSM 22312, 75.73% for *E. coli* CL1 and 100% for *E. coli* CL2 within 72 h.

The survival rate of *G. mellonella* larvae increased when infected with *E. coli* strains pre-treated with berberine at ½ and ¼ MIC. This effect was both concentration and strain-dependent. The increased survival rate of larvae inoculated with *E. coli* strains pre-treated with 1/2 berberine MIC is statistically significant for *E. coli* ATCC 11775, *E. coli* DSM 22312 and CL2. Meanwhile, for 1/4 MIC pre-treatment, a reduction was observed only for *E. coli* ATCC 11775. As regards the co-incubation assay, in all UPEC groups treated with 2 MIC berberine, a significant reduction in mortality for all strains was observed.

### 2.5. Effects of Berberine Chloride on E. coli Recovery (CFU/mL) from G. mellonella Haemolymph

**Figure 5:**
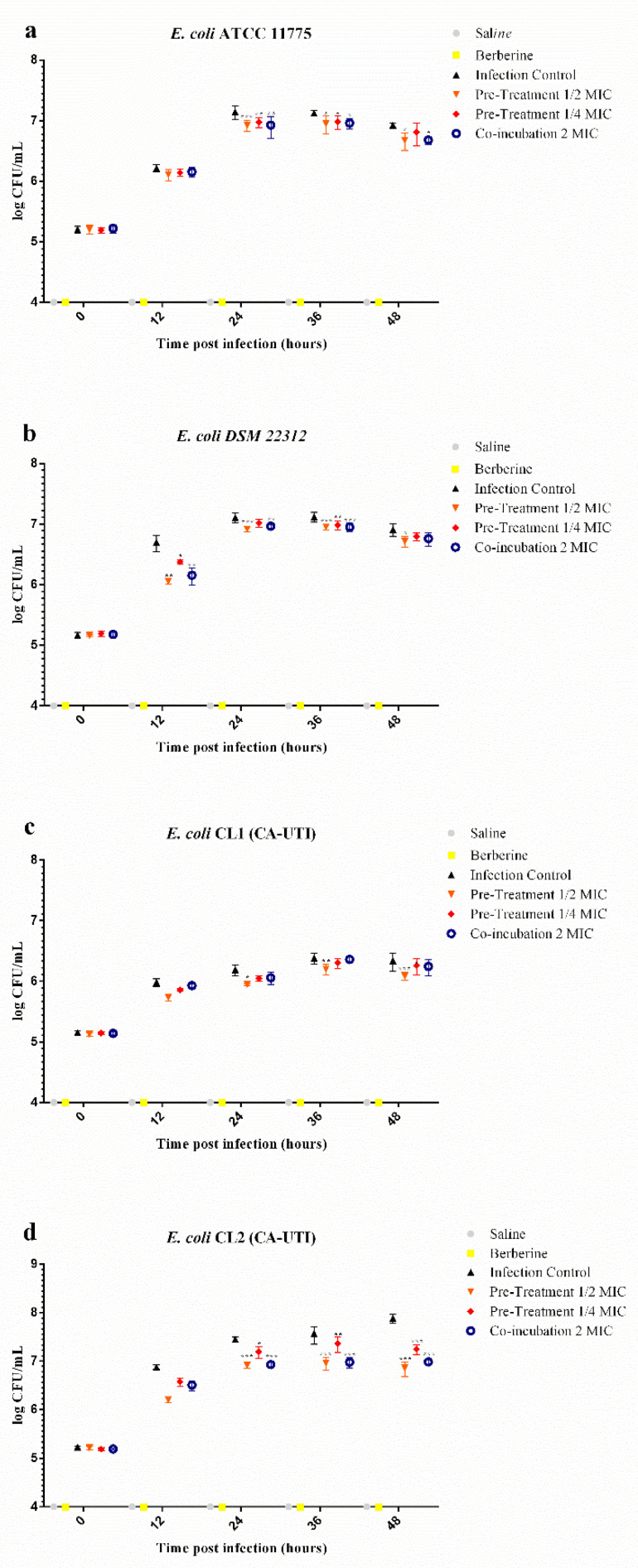
CFU/mL of *E. coli* strains recovered from *G. mellonella* larvae haemolymph infected with 10^5^ CFU/larvae and treated with berberine at different concentration. Light grey circles: saline inoculated larvae; Yellow squares: Berberine control; Black triangles: *E. coli* 10^5^ CFU/larvae inoculated; Orange triangles: *E. coli* pre-treated berberine 1/2MIC infected larvae; Red squares: *E. coli* berberine 1/4MIC pre-treated infected larvae; blue circles: berberine co-incubated infected larvae. (**a**) *E. coli* ATCC 11775. (**b**) *E. coli* DSM 22312. (**c**) *E. coli* CL1 (CA-UTI). (**d**) *E. coli* CL2 (CA-UTI). Data expressed as the mean ± standard deviation (log10 CFU/mL of hemolymph) of three independent experiments. Statistical analysis was performed using the two-way ANOVA with Bonferroni multiple comparison post-test. *** *p* < 0.001, ** *p* < 0.01, * *p* < 0.05.

*E. coli* recovery (CFU/mL) from *G. mellonella* haemolymph revealed a lower bacteria cell count for all tested strains in both pre-treated and co-incubation experiments at all time points (Figure 5 and Table 3). In larvae haemolymph infected with *E. coli* pre-treated with 1/2 MIC berberine, bacterial cells recovered were significantly reduced at 24 h, 36 h and 48 h for *E. coli* ATCC 11775, CL1 and CL2, while at 12 h, 24 h, 36 h and 48 h for *E. coli* DSM 22312.

As regards larvae inoculated with the pre-treated strains at 1/4 MIC, a significant reduction was found at 24 h and 36 h for *E. coli* ATCC 11775, 12 h and 36 h for *E. coli* DSM 22312, 48 h for *E. coli* CL1 and 24 h, 36 h and 48 h for *E. coli* CL2. In the co-incubation group, a significant reduction in the pathogen count was observed at 24 h, 36 h and 48 h for *E. coli* ATCC 11775 and CL2, 12 h, 24 h, and 36 h for *E. coli* DSM 22312. In all non-infected larval groups, *E. coli* was not detected.

### 2.6. Effects of Berberine Chloride on the Enumeration of G. mellonella Hemocytes

**Figure 6:**
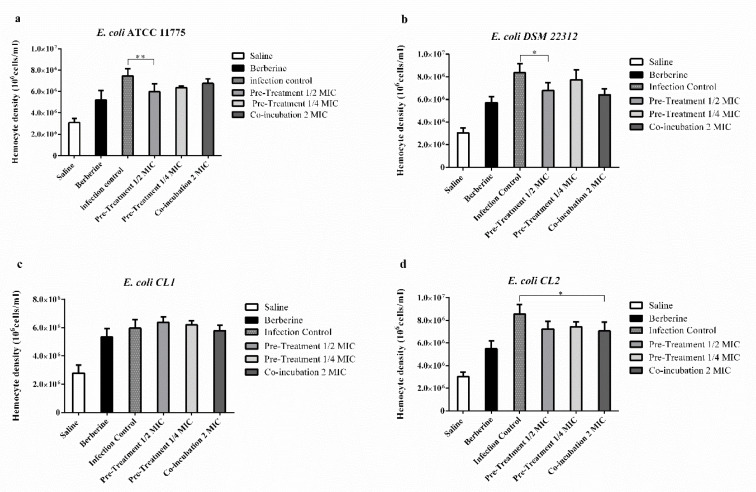
Hemocyte count of *G. mellonella* larvae infected with *E. coli* 10^5^ CFU/larvae and treated with berberine. Larvae treated with: saline (White histograms) or berberine. (Black histograms). Dark grey histograms with small squares: larvae infected with *E. coli* strains. Light grey histograms: larvae infected with *E. coli* pre-treated with 1/2 MIC and with 1/4 MIC berberine. Dark grey histograms: larvae infected and co-incubated with berberine. (**a**) *E. coli* ATCC 11775. (**b**) *E. coli* DSM 22312. (**c**) *E. coli* (CA-UTI) CL1. (**d**) *E. coli* CL2 (CA-UTI). Results are expressed as cells/mL. The bars represent means ± SD of three independent experiments performed in triplicate. Statistically significant differences, determined by one-way analysis of variance ANOVA (* indicates a significant difference at *p* < 0.05 versus control/untreated, ** indicates a significant difference at *p* < 0.01 versus control/untreated).

The effect of berberine on *G. mellonella* immune response was evaluated by hemocyte enumeration. After 48 h of exposure to berberine alone, the hematocyte count increased, but not significantly. In larvae infected with *E. coli* ATCC 11775 and *E. coli* DSM 22312, a significant reduction in the haematocytes count was observed in the pre-incubated group (1/2 berberine MIC) compared to untreated larvae. Meanwhile, in larvae infected with *E. coli* CL2, a significant reduction of the co-incubated group compared to the infected group was found (Figure 6).

## 3. Discussion

Herbs used in traditional medicine have increasingly gained interest as their mechanisms differ from commonly used antibiotics and they could be used as complementary agents to prevent or co-treat UTIs [37].

The emergence of antimicrobial resistance demands novel antibiotic-sparing therapeutic approaches for UTIs treatment and prevention [58].

In this context, the berberine chloride mechanism towards bacteria is well known. It targets the principal physiological functions of the bacteria cell, including inhibition of DNA duplication, RNA transcription, and protein biosynthesis in bacterial cells by inhibition of enzyme activities and modifications of the surface structure of bacterial cell walls [49,59].

Due to this broad-spectrum mechanism of action, Jin et al. demonstrated that the in vitro exposure of several bacterial generations to berberine (including *E. coli*), makes the resistance onset to this substance extremely difficult because of the lack of live and metabolically active clones [60].

Moreover, berberine is also able to influence the synthesis and expression of PAP (pyelonephritis-associated pili) *fimbriae* UPEC [47,49]. As adhesion virulence factors, these peculiar *fimbriae* play an essential role in UPEC kidneys colonization [61]. Therefore, the mechanism of action of berberine on *E. coli* strains has been extensively investigated by proteomic and transcriptomic studies [46,62,63].

Our data demonstrated that, although the MIC range of the four *E. coli* strains tested is between 2048 and 1024 µg/mL (Table 1), the in vitro growth curves study at sub-inhibiting doses (1/2 and 1/4 MIC) revealed a strain-dependent action. Indeed, the DSM 22312 strain, with a MIC value (2048 µg/mL), showed a higher susceptibility to berberine as concerned inhibition rates and the delay in reaching the exponential growth phase (Figure 1b and Table 2). On the other hand, the effect of berberine on the slope of the curves (Hill slope) was more evident in the clinical strain CL2, although it had a MIC value of 2048 µg/mL (Figure 1d and Table 2).

These data are in agreement with the work carried out by Sun et al. in which the inhibiting action of berberine on the growth and biofilm formation of multidrug-resistant *E. coli* strains was detected [50].

The in vitro results of the ATCC 5637 HTB-9 cell line demonstrated that pre-treatment with berberine at the sub-inhibitory concentration tested (1/2 MIC) significantly decreases the adhesion and invasion of the tested *E. coli* strains to bladder cells (Figure 2 and Figure 3). However, berberine co-incubation did not significantly reduce tested strains of adhesive and invasive ability.

Moreover, for the first time, the in vitro berberine anti-invasive activity was reported (Figure 3). If this activity can be confirmed by in vivo evidence, it would have significant therapeutic implications, given that when UPEC invade the urinary tract epithelial host cells, they escape from host defences and antibiotic treatments. Moreover, the formation of stable intracellular bacterial populations can create reservoirs for chronic UTI [26].

As regards the in vitro berberine anti-adhesive activity, our results showed a strong influence of several experiment conditions such as the cell lines used, the concentration and the exposure times. These can explain the difference between our findings and those reported by Sun et al. in a similar in vitro experiment [47].

This scenario led us to evaluate whether the in vivo model *G. mellonella* reflected the results obtained by the in vitro antibacterial, anti-adhesive and anti-invasive action of berberine against four UPEC strain on ATCC 5637 HTB-9 bladder cells. To this end, the *larvae* survival, the bacterial cell count recovered and hematocyte count after infection were carried out. Furthermore, to our knowledge, the preliminary in vivo data presented in this study represent the first evidence about the action of berberine in UPEC infection. The low berberine toxicity made this model suitable for experiments on antimicrobial activity and larval survival, so the 2 MIC concentration was chosen for in vivo *G. mellonella* co-incubation assay to assess *larvae* survival and bacteria cell recovery.

The protective action of berberine towards infected *larvae* was detected both in pre-treatment and co-incubation experiments (Figure 4).

On the other hand, it is not surprising that the data obtained from these insect models are in agreement with the in vivo mouse model reported by Pierpaoli et al., that aim to evaluate berberine activity by intraperitoneal administration against *E. coli* sepsis [64].

The *larvae* protective effect observed in co-incubated groups (Figure 4) could be due to berberine anti-inflammatory action on lipopolysaccharide (LPS) [65]. A study reported by Mahadavi et al. demonstrated that the administration of berberine protects mice from LPS-induced abortion by modulating the inflammatory process and immune response [66]. Treatment with berberine increased the survival rate of mice with LPS-induced endotoxemia, and this effect was due to its high affinity for the TLR4/ MD-2 receptor with the pro-inflammatory cascade block [67].

The results obtained from *larvae* haemolymph CFU/mL *E. coli* counting give us the chance to explore the dynamics of the infection. Indeed, *E. coli* counts were significantly reduced by both treatments (Figure 5 and Table 3), and this would seem to suggest that berberine, through its antimicrobial activity and/or by interfering with the adhesion of bacterial strains, could reduce the virulence of the strain allowing the *larvae*’s innate immune system to respond infection more effectively.

Additionally, a hematocytes count increase was observed, although not statistically significant, in berberine-only-treated larvae. Hematocytes play an important role in *G. mellonella* immune response to infection, so this increase could act in combination with the antimicrobial properties of berberine to prevent larvae death [68]. Statistically significant reductions in hematocyte counts were observed in pre-incubated 1/2 mic and co-incubated groups with berberine 48 h after infection. This is well correlated with the results of the CFU/mL *E. coli* enumeration test 48 h after infection and may be attributed to successful hematocytes phagocytosed the pathogen during the early stages of the infection.

In light of the numerous pieces of evidence reported in this paper, we can state that the in vivo model *G. mellonella* was able to provide promising preliminary results about berberine activity versus UPEC pathogenetic mechanism, providing new opportunities for berberine use in UTI treatment and prevention.

Moreover, besides the numerous therapeutic potentials of berberine [8,69,70], toxicological effects have also been reported. As published by Sing et al., the LD50 values for berberine are very variable (2600 to 23 mg/Kg) and strongly depend both on the amount of active substance present in the compound tested (i.e., extract or pure substance) and on the route of administration as well as the type of organism [71].

Several studies demonstrating the in vitro synergistic and protective effect between berberine and antimicrobials have already been published [72,73,74,75]. The use of this substance as a therapeutic complement in human bacterial infections, especially against relevant strains of *E. coli*, although not included among the objectives of this study, deserves to be investigated in depth using *G. mellonella* as in vivo screening pre-clinical model. Moreover, the concentration tested in this study may be very high for future clinical studies, so will be necessary to evaluate a possible synergistic combination between berberine and other antimicrobials by *G. mellonella* in vivo model. This is to reduce the possible side effects related to its administration, especially in those patients who have a complicated medical history or chronic medical conditions (i.e., those most likely to receive a urinary catheter). In this scenario, promising results were obtained by Pierpaoli et al. in the treatment of *E. coli*-induced sepsis by intraperitoneal administration of berberine associated with imipenem on mice [64].

## 4. Materials and Methods

### 4.1. Chemicals and Culture Medium

Berberine chloride, MW 371.81 (European Pharmacopoeia (EP) Reference Standard; purity not less than 98%), sodium chloride, potassium chloride, tris–HCl, EDTA, sodium citrate, Brain Heart Infusion Agar and Broth (BHI), Mueller Hinton broth (MH), Brilliance *UTI* agar, Luria–Bertani (LB) broth and Triton X-100 were all purchased from Sigma-Aldrich (Milan, Italy). RPMI-1640 Medium, fetal bovine serum, penicillin/streptomycin (p/s), L-glutamine 200 mM and Dulbecco’s phosphate-buffered saline (DPBS) were purchased from Gibco (Thermo Fisher Scientific, Waltham, MA, USA). Trypsin EDTA 1X was purchased from Corning (Corning, NY, USA).

### 4.2. Bacterial Strains, Cell Line and Larvae

*Escherichia coli* ATCC 11775 (isolated from urine) was purchased from the American Type Culture Collection (Rockville, MD, USA), *Escherichia coli* DSM 22312 (isolated from UTI) was purchased from DSMZ-German Collection of Microorganisms and Cell Cultures GmbH (Braunschweig, Germany).

*Escherichia coli* CL1 (CA-UTI) and *Escherichia coli* CL2 (CA-UTI) were isolated from long-therm catheters carriers’ patients affected by chronic urinary infections. Clinical strains were previously identified by biotyping with GN-ID A and GN-ID B Microgen^®^ gallery using Identification Software Microgen^®^ version 2.0.8.33 (Kairosafe Srl Sistiana TS Italy). All strains were cultured in Brain Heart Infusion Agar Broth aerobically with shaking at 37 °C.

Human bladder carcinoma (ATCC 5637 HTB-9) cell line was purchased from the American Type Culture Collection. The cell line was cultured in RPMI-1640 medium, supplemented with 10% fetal bovine serum, 1% of l-glutamine and 1% penicillin/streptomycin (p/s), in 95% humidified air, with 5% CO_2_ at 37 °C.

*G. mellonella larvae* were purchased from SA.GI.P. s.a.s. (Ravenna, Italy). The diet for rearing larvae consists of cornmeal, soy meal, sorbitol, sugar, honey and beeswax. The company supplies live wax worm *larvae* of appropriate size and untreated. These characteristics allow the *larva*, at room temperature, to maintain a greater consistency useful for handling in laboratory practice. They were stored at 15 °C in the dark and were used within seven days of receipt.

### 4.3. Berberine MIC Determination

Evaluation of in vitro berberine chloride MIC was estimated by the microdilution method. Inoculum preparation, standardization and preparation together with culture media used (CHMH) and incubation conditions were performed according to CLSI guidelines for antimicrobial susceptibility test (CLSI M100) [76]. Berberine was dissolved in DMSO 68.2 mg/mL in DMSO to make a stock solution and before use diluted appropriately in deionized water and filtered through a 0.22 µm Millipore filter.

Berberine concentrations tested were from 2048 to 0.5 μg/mL. The 96-well microplates were incubated at 37 °C for 24 h, and MIC was defined as the lowest concentration of compounds at which bacterial growth was inhibited. The highest DMSO concentration that has not interfered with bacteria growth was 3% (*v*/*v*) (data are not shown).

### 4.4. Influence of Berberine on Bacterial In Vitro Growth

The influence of berberine chloride on in vitro growth curves was performed according to Sun et al., with some changes [50]. Briefly, bacterial suspensions were standardized to the concentration of 1 × 10^8^ CFU/mL by spectrophotometric determination (OD 600). A series of dilutions were prepared to obtain a final concentration of 5 × 10^5^ CFU/mL in MH broth. Berberine was added in two different concentrations (1/2 MIC and 1/4 MIC). Inoculated bottles were incubated aerobically with shaking at 37 °C; 100 µL of each sample was taken every 60 min for 24 h and analyzed by viable bacteria count. For this purpose, serial 10-fold dilutions in saline solution (NaCl 0.85% *w*/*v*) were performed, and each dilution was spread on MH plates agar.

### 4.5. In Vitro Bacterial Grown Curve Analysis

The analysis of the bacterial growth curves was performed according to the method proposed by Nicolosi et al., [77]. The percentage of inhibition of bacterial growth (% inhibition) and the time delay (expressed in hours) in reaching the exponential growth (∆X_CFU50_) for each strain was calculated by analyzing the in vitro bacterial growth curves.

Data from all the strains tested were analyzed with Sigmoidal dose–response (variable slope) function by Prism GraphPad Software 6 (GraphPad Software, California, CA, USA).

The Hillslope parameter represents the steepness of the curve. Additionally, the curve ∆ hillslope representing the steepness difference between the tested strains and the control was calculated according to the following formula:

∆Hillslope = (Strain hillslope at 1/2 or 1/4 MIC berberine) − (Strain Hillslope Control).

### 4.6. Effect of Berberine on E. coli Strains Adhesion and Invasion to Human Bladder Cells ATCC 5637 HTB-9: Co-Incubation and Pre-Treatment Experiments

*E. coli* strains adherence and internalization on the human bladder carcinoma (ATCC 5637 HTB-9) cell line was determined according to the method proposed by Mohanty et al. with some modifications [78]. All media utilized for the assay were free of antibiotics and serum. Overnight bacterial suspension of *E. coli* strains cultured in BHI broth at 37 °C and berberine at the concentration of 1/2 MIC, and 1/4 MIC was used for pre-treatment and co-incubation experiments.

In the pre-treated assay the *E. coli* bacterial suspensions were diluted at the concentration of 10^8^ CFU/mL in MH broth and incubated at 37 °C with berberine chloride at the concentration of 1/2 MIC or 1/4 MIC for four hours.

For co-incubation treatment, bacterial suspensions of *E. coli* strains were diluted at the concentration of 10^8^ CFU/mL in MH broth and incubated at 37 °C without berberine chloride for four hours.

Afterward, 100 μL of bacterial suspensions for each strain were washed twice with Phosphate-Buffered Saline (PBS) (Gibco), were normalized using optical density (OD600) and then resuspended in RPMI medium. Serial dilutions of the bacterial suspensions were plated in MH agar plate to determine the bacterial load initially inoculated into the cells.

Cell monolayers were grown to approximately 95% confluence in 6-well tissue culture plates (Corning), washed three times with Dulbecco’s phosphate-buffered saline (DPBS) and infected with 2 mL per well of the bacterial suspension. Cells were infected at a multiplicity of infection (MOI) of approximately 30 bacteria per cell (∼4 × 10^7^ bacteria). The co-incubation groups were simultaneously treated with berberine added to the cell medium at 1/2 MIC or 1/4 MIC concentration immediately after infection with the bacterial suspensions. After incubation in 5% CO_2_ at 37 °C for 2 h, the cells were washed three times with DPBS to remove non-adherent bacteria. For the adhesion test, cells were scraped off in 1 mLof PBS, and the total bacterial count was determined by plate count method. Serial dilutions were plated on MH agar and incubated at 37 °C for 24 h to determine bacterial CFU/mL. In the invasion assay, fresh medium containing 100 μg/mL of gentamicin was added to kill adhered extracellular bacteria. After an additional incubation (2 h at 37 °C, 5% CO_2_), the monolayers were washed three times with DPBS, and epithelial cells were lysed by addition of 100 μLof 0.25% trypsin–EDTA and 900 μL of 0.05% Triton X-100 for 5 min. The total bacterial count was evaluated as described for adhered bacteria.

### 4.7. G. mellonella Infection: Co-Incubation and Pre-Treatment Assays

*Larvae* were grouped according to their weight (from 300 to 350 mg), light-coloured and free of dark spots and/or cuticle pigments. Preliminary experiments were conducted to assess the toxicity of berberine chloride by injection of serial dilutions of the test substance (data not shown).

For bacteria inoculum preparation, overnight bacterial suspensions of *E. coli* strains cultured in BHI broth at 37 °C and berberine at the concentration of 1/2MIC or 1/4 MIC were used for pre-treatment experiments, while the concentration 2 MIC was used for co-incubation assays.

To set up an optimal inoculum concentration (CFU/mL), time-killing curves were made by inoculating *larvae* with 20 µL of bacterial suspensions standardized by plate count in a concentration range from 1 × 10^2^ to 1 × 10^8^ CFU/*larvae* (data not shown). From this data, an infectious dose of 10^5^ CFU/*larvae* was chosen for all subsequent experiments.

As described before, for pre-treated assay bacterial suspensions of *E. coli* strains were diluted at the concentration of 10^8^ CFU/mL in MH broth and incubated at 37 °C with berberine chloride at the concentration of 1/2 MIC or 1/4 MIC for four hours. While for the co-incubation treatment, bacterial suspensions of *E. coli* strains were diluted at the concentration of 10^8^ CFU/mL in MH broth and incubated at 37 °C without berberine chloride for four hours. Afterward, the bacterial suspensions were centrifuged at 5000 rpm for two minutes.

The bacterial pellet was washed 3 times with DPBS and resuspended in the same medium. The suspensions were normalized by plate count for colony-forming units (CFU/mL) determination. Bacteria inoculation was performed using a syringe pump with a 26-gauge half-inch syringe into the right hemocoel at the last proleg after swabbing the area with ethanol 70% immediately before treatment.

*Larvae* used for the co-incubation experiment were injected with 2 MIC berberine solution fifteen minutes after the bacterial infection. After injections, twenty *larvae* for each experiment were incubated in a Petri dish at 37 °C, and survival percentage was determined in 72 h. *Larvae* were considered dead when there was no movement in response to stimuli and an extensive melanization was observed.

### 4.8. G. mellonella Haemolymph Collection, E. coli Strains Recovery (CFU/mL) and Haemocytes Enumeration

Haemolymph collection was performed according to the method proposed by Jorjão et al. [79] for all tested groups.

For *E. coli* strains recovery, the *haemolymph* of three *larvae* for each group was collected aseptically, diluted in LB medium and plated onto Brilliance UTI agar, a chromogenic medium for differentiation of all the main microorganisms that cause urinary tract infections (UTIs). For bacterial CFU/mL determination, only pink colony (*E. coli*) were taken into account [80].

Hemocytes count was performed on *G. mellonella larvae* (three *larvae* per group), at 48 h after infection according to Jorjão et al. Briefly, after collection, haemolymph was centrifuged, washed and resuspended in Insect Phosphate Saline (IPS) and finally tested for cell viability by exclusion test with trypan blue.

### 4.9. Statistical Analysis

Data are expressed as mean ± standard deviation (SD) for three replicates of three independent experiments (i.e., biological and technical triplicates). Prism GraphPad Software 6 was used for all statistical analyses. The goodness of Fit for Sigmoidal dose–response (variable slope) function was verified by R squared values > 0.99 for all the data tested. Differences between groups in adhesion and invasion to human ATCC 5637 HTB-9 cells and hematocytes count assay were assessed by one-way ANOVA, followed by Bonferroni multiple testing correction. Survival curves of *G. mellonella* analysis were performed by the Log-rank Mantel–Cox test. Results were considered statistically significant at *p* < 0.05. A two-way ANOVA with Bonferroni multiple comparison post-test was performed to compare the differences between control and treatment in quantification bacteria CFU/mL *hemolymph* assay.

## 5. Conclusions

To sum up, the in vitro and in vivo studies on berberine versus UPEC strains demonstrated once-again the efficacy and reliability of the *G. mellonella* model evaluation for toxicity, survival and bacterial cell recovery studies of plant extract employed as a therapeutic complement for human bacterial infections.

In vitro berberine pre-treatment was able to decrease the adhesive and invasive UPEC ability. Meanwhile, the in vivo model gives us the chance to explore the dynamics of the infection, demonstrating the effect of both pre-treatment and co-incubation berberine activities.

## Figures and Tables

**Table 1:** Minimum Inhibitory Concentrations (MIC) of *E. coli* strains to berberine chloride (μg/mL).

Strains	Berberine
*E. coli* ATCC 11775	1024
*E. coli* DSM 22312	2048
CL1 (CA-UTI)	1024
CL2 (CA-UTI)	2048

**Table 2:** In vitro bacterial grown curves analysis. Control/Untreated (CNT); standard deviation (SD); Ytop (the maximum value of CFU/mL); X_CFU50_ (The time delay in reaching the exponential growth phase); HillSlope (the steepness of the curve).

	*E. coli* ATCC 1775		*E. coli* DSM 22312				*E. coli* CL1			*E. coli* CL2		
	CNT	¼ MIC	½ MIC	CNT	¼ MIC	½ MIC	CNT	¼ MIC	½ MIC	CNT	¼ MIC	½ MIC
Goodness of Fit R^2^	0.998	0.998	0.998	0.997	0.995	0.999	0.996	0.997	0.998	0.997	0.998	0.999
Y_Top_ (CFU/m)	9.66 × 10^8^	8.45 × 10^8^	6.12 × 10^8^	1.03 × 10^9^	7.19 × 10^8^	5.87 × 10^8^	9.59 × 10^8^	7.75 × 10^8^	5.49 × 10^8^	1.04 × 10^9^	8.58 × 10^8^	7.79 × 10^8^
Ytop ± S.D (CFU/mL)	1.98 × 10^7^	1.54 × 10^7^	2.11 × 10^7^	3.00 × 10^7^	2.92 × 10^7^	9.00 × 10^6^	2.95 × 10^7^	2.17 × 10^7^	1.08 × 10^7^	3.30 × 10^7^	2.36 × 10^7^	1.13 × 10^7^
Inhibition (%)	/	12.47	36.61	/	30.29	43.03	/	19.25	42.72	/	17.75	25.35
X_CFU50_ (h)	5.87	8.58	9.03	4.99	7.63	8.76	5.05	6.26	6.86	5.79	6.69	8.42
X_CFU50_ ± S.D. (h)	0.35	0.3	0.28	0.57	0.5	0.16	0.62	0.44	0.24	0.62	0.5	0.2
∆X_CFU50_ (h)	/	2.71	3.16	/	2.64	3.77	/	1.21	1.81	/	2.63	0.9
HillSlope	0.24	0.24	0.27	0.25	0.28	0.3	0.24	0.25	0.31	0.22	0.19	0.20
HillSlope ± S.D	0.039	0.037	0.043	0.06	0.081	0.032	0.064	0.054	0.049	0.054	0.036	0.018
∆ HillSlope	/	0	0.03	/	0.03	0.05	/	0.01	0.07	/	−0.03	−0.02

**Table 3:** Log units reduction and significant differences determined by two-way ANOVA with Bonferroni multiple comparison post-test.

		*E. coli* ATCC 1175		*E. coli* DSM 22318		*E. coli* CL1		*E. coli* CL2	
		Log Units	*p* Value	Log Units	*p* Value	log Units	*p* Value	log Units	*p* Value
time post infection 12 h	Infection Control vs. Pre-Treatment 1/2 MIC	0.1093	>0.9999	0.6462	0.0014	0.2492	0.2989	0.6700	0.5716
	Infection Control vs. Pre-Treatment 1/4 MIC	0.0767	>0.9999	0.3220	0.0406	0.1255	>0.9999	0.3000	>0.9999
	Infection Control vs. Co-incubation MIC	0.0612	>0.9999	0.5442	0.0032	0.0524	>0.9999	0.3689	>0.9999
time post infection 24 h	Infection Control vs. Pre-Treatment 1/2 MIC	0.2225	0.001	0.1888	0.0002	0.2422	0.0329	0.5333	0.0002
	Infection Control vs. Pre-Treatment 1/4 MIC	0.1706	0.0083	0.0906	0.0675	0.1411	0.2733	0.2519	0.0239
	Infection Control vs. Co-incubation MIC	0.2191	0.0011	0.1428	0.0032	0.1304	0.3411	0.5190	0.0002
time post infection 36 h	Infection Control vs. Pre-Treatment 1/2 MIC	0.1761	0.0101	0.1708	0.0004	0.1849	0.005	0.6078	<0.0001
	Infection Control vs. Pre-Treatment 1/4 MIC	0.1462	0.0323	0.1364	0.0036	0.0807	0.3198	0.2036	0.0096
	Infection Control vs. Co-incubation MIC	0.1648	0.0156	0.1676	0.0005	0.0214	>0.9999	0.5896	<0.0001
time post infection 48 h	Infection Control vs. Pre-Treatment 1/2 MIC	0.2486	0.0432	0.1964	0.0177	0.2520	0.0011	1.0291	<0.0001
	Infection Control vs. Pre-Treatment 1/4 MIC	0.1105	0.5842	0.1163	0.2028	0.0791	0.4485	0.6337	<0.0001
	Infection Control vs. Co-incubation MIC	0.2425	0.0485	0.1491	0.0758	0.0968	0.2572	0.9015	<0.0001
